# Late-Onset Hemorrhagic Pericardial Effusion and Cardiac Tamponade Associated With Immune Checkpoint Inhibitors: Case Report and Literature Review

**DOI:** 10.7759/cureus.42867

**Published:** 2023-08-02

**Authors:** Aleksan Khachatryan, Joel M Alejandro, Robert D Chow, Reyaz U Haque, Jamal A Mikdashi

**Affiliations:** 1 Internal Medicine, University of Maryland Medical Center Midtown Campus, Baltimore, USA; 2 Cardiology, University of Maryland Medical Center Midtown Campus, Baltimore, USA; 3 Rheumatology, University of Maryland Medical Center Midtown Campus, Baltimore, USA

**Keywords:** pericarditis, pericardiocentesis, pembrolizumab, diffuse large b cell lymphoma, pericardial disease, hemorrhagic pericardial effusion, tamponade, immune-related adverse events, immune checkpoint inhibitors

## Abstract

Immune checkpoint inhibitors (ICIs) are monoclonal antibodies that target T lymphocytes and stimulate the immune system. However, the use of ICIs is associated with immune-related adverse events (irAEs). Pericardial disease is a cardiovascular irAEs that can present as cardiac tamponade. The precise mechanisms underlying pericardial complications are not fully understood. Late-onset hemorrhagic pericardial effusion associated with ICIs is quite rare; the mechanism and predisposing factors are yet to be determined.

This case report describes a patient with diffuse large B-cell lymphoma (DLBCL) who received pembrolizumab for 390 days and subsequently developed cardiac tamponade caused by hemorrhagic pericardial effusion. The purpose of this report is to raise awareness about the occurrence of late-onset cardiac tamponade and provide a summary of available data on patients who experienced hemorrhagic pericardial effusion during ICI treatment.

## Introduction

Immune checkpoint inhibitors (ICIs) are antibodies, and pembrolizumab specifically targets the programmed cell death receptor 1 (PD-1), activating T lymphocytes against cancer cells. Diffuse large B-cell lymphoma (DLBCL) is the most common subtype of non-Hodgkin lymphoma (NHL). While ICIs have been approved for treating relapsing, progressive, or refractory Hodgkin lymphoma, recent studies have shown promising results for pembrolizumab in the treatment of relapsed/refractory DLBCL [1]. The use of ICIs is expanding not only in different types of advanced cancer management but also as a primary treatment for specific cancers due to their favorable outcomes. However, the widespread use of ICIs has also led to increased identification of immune-related adverse events (irAEs). The most common cardiovascular irAEs include myocarditis, pericardial disease, heart failure, dyslipidemia, myocardial infarction, and cerebral arterial ischemia [2]. ICI use is also associated with major adverse cardiac events [3]. One suggested mechanism of pericardial irAEs involves pericardial inflammation by ICI-stimulated cytotoxic T cells. Manifestations of pericardial disease include pericarditis, pericardial effusion, and, in rare instances, cardiac tamponade [4]. The typical onset of pericardial disease associated with pembrolizumab use occurs within the first three weeks of treatment [5]. Cardiac tamponade resulting from hemorrhagic pericardial effusion is an extremely rare occurrence, with only a few cases documented in the literature. Further research is still required to elucidate the underlying mechanisms and predisposing factors of hemorrhagic pericardial effusion.

We conducted a review of the existing literature to identify the factors associated with the development of malignancy-negative hemorrhagic pericardial effusion, aiming to enhance understanding of this complication. The literature search ranged from March 1, 2023, to July 5, 2023, focusing specifically on cases of hemorrhagic pericardial effusion associated with the use of ICIs. The search terms we used included “hemorrhagic pericardial effusion associated with Immune checkpoint inhibitors”, “serosanguinous pericardial effusion and immune checkpoint inhibitors”, and “cardiac tamponade and immune checkpoint inhibitors”. A total of 11 case reports and literature reviews, published between 2015 and 2023, were analyzed.

We present this case to highlight the potential for ICI-mediated late-onset irAEs manifesting as cardiac tamponade, a life-threatening complication that necessitates prompt recognition and intervention. This case was presented as a poster at the Maryland Chapter American College of Physicians annual meeting on May 11, 2023.

## Case presentation

A 44-year-old female with a history of congestive heart failure with recovered ejection fraction (EF) (due to chemotherapy), postural tachycardia syndrome, asthma, stage IV DLBCL on pembrolizumab presented with dyspnea and chest tightness in November 2022. The last dose of pembrolizumab was given 12 days prior to this presentation. Notably, she denied syncope, dizziness, paroxysmal nocturnal dyspnea (PND), orthopnea, or leg swelling. 

The patient's oncological history was significant for DLBCL diagnosed in April 2019. The initial treatment involved six cycles of rituximab, cyclophosphamide, doxorubicin, prednisone, and vincristine (R-CHOP) chemotherapy, completed in August 2019. In October 2019, a 35-day course of proton therapy was started to address residual disease in the mediastinum and right superior lung field. The CT scan later showed active disease, and the patient received two cycles of rituximab, ifosfamide, carboplatin, and etoposide (RICE) chemotherapy in January 2020, followed by haploidentical stem cell transplantation in March 2020. In September 2020, she received chimeric antigen receptor (CAR) T-cell therapy for recurrent DLBCL, after one cycle of rituximab and polatuzumab therapy, leading to remission for one year. However, the core biopsy of the left lung mass confirmed recurrent lymphoma, and pembrolizumab was initiated in October 2021. The patient had been on pembrolizumab for 390 days, with follow-up PET/CT scans demonstrating no evidence of active lymphoma

From a cardiovascular perspective, the patient was diagnosed with chemotherapy-induced cardiomyopathy, with the lowest left ventricular EF of 30-35%. Subsequent echocardiography in March 2022, while the patient was on pembrolizumab, revealed an improved EF of 50-55%, and no signs of pericardial effusion.

The initial vital signs were as follows: temperature of 37.5°C, heart rate of 75 beats per minute, blood pressure of 118/81 mmHg, respiratory rate of 20 breaths per minute, and oxygen saturation of 96% on room air. The physical examination was negative for murmurs rub, gallops, jugular venous distension, or peripheral edema.

**Figure 1:**
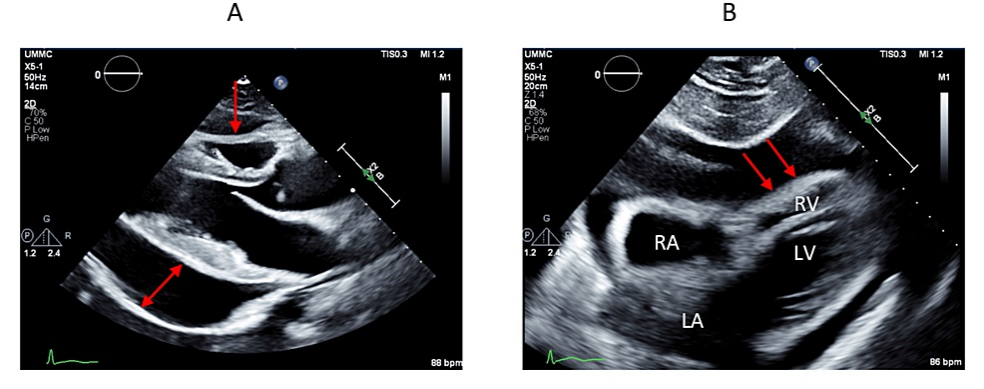
Echocardiography demonstrating tamponade physiology A: demonstrates pericardial effusion posterior to the left ventricle (indicated by the red double arrow) and early diastolic RV collapse (indicated by the red arrow) on the parasternal long-axis view. B: highlights RV collapse during early diastole (indicated by the red arrows) on the subxiphoid view. RA: right atrium; RV: right ventricle; LA: left atrium; LV: left ventricle

Troponin levels were not elevated and ECG was unremarkable. Echocardiography demonstrated normal EF of 60-65%, large pericardial effusion, and intermittent early diastolic right ventricular and right atrial collapse concerning for tamponade physiology as depicted in Figure 1.

The cardiac PET/CT scan was negative for new skeletal, nodal, or extranodal involvement, demonstrating pericarditis-type of changes. Pericardiocentesis removed 460 ml of hemorrhagic fluid with numerous red blood cells (RBCs) and lymphocytic predominance (Table 1).

**Table 1:** Pericardial fluid analysis WBC: white blood cell; RBC: red blood cell; LDH: lactate dehydrogenase

Pericardial fluid analysis	Results	Reference range
WBC	1,520 mm3	0 - 0 mm3
RBC	880,000 mm3	0 - 0 mm3
Polys	20 %	N/A
Lymphocytes	44 %	N/A
Monocytes	36 %	N/A
Total protein	6 g/dL	N/A
Glucose	74 mg/dL	N/A
LDH	2,881 units/L	N/A
Appearance	Bloody	Clear

Pericardial fluid analysis was negative for gram stain and bacterial cultures, acid-fast bacillus (AFB) smear, and tuberculosis (TB) culture. Similarly, the upper respiratory viral panel results showed no presence of viral infections. The cytology was negative for malignant cells and flow cytometry was negative for B-cell lymphoma. The thyroid stimulating hormone (TSH) level was normal. Additionally, the most recent antinuclear antibody (ANA) titers were normal, suggesting the absence of autoimmune disorders.

The pembrolizumab was held, and methylprednisolone 1mg/kg was started, which was later switched to prednisone. As a result, the pericardial effusion resolved, and the drain was removed on the third day. The patient was discharged with no recurrence of pericardial effusion on follow-up echocardiography.

## Discussion

The diagnosis of ICI-induced hemorrhagic pericardial effusion is typically a diagnosis of exclusion. It is important to note that for patients with a history of malignancy presenting with late-onset pericardial effusion and tamponade, the likelihood of metastatic involvement of the pericardium as the underlying cause is high in differentials [6]. Furthermore, other potential etiologies to consider include infectious causes, as well as pericardial disease induced by radiation or chemotherapy. Negative pericardial fluid cytology, in conjunction with normal flow cytometry results (with the latter being particularly relevant for lymphoma diagnosis), significantly diminishes the possibility of malignant pericardial effusion. Additionally, PET/CT scan results revealed no signs of neoplastic pericardial involvement. Notably, the pericardial fluid analysis was negative for bacterial stains and growth, including AFB, essentially ruling out infection as an underlying cause of the hemorrhagic pericardial effusion. Considering that the patient underwent chemotherapy approximately three years ago, it is highly unusual for the pericardial effusion to be induced by this therapy after such a prolonged period of time. While pericardial effusions may manifest months to years after radiotherapy, the occurrence of cardiac tamponade resulting solely from proton therapy after three years is unlikely, given that pericardial effusion is a relatively uncommon complication of modern radiotherapy techniques, such as proton radiotherapy. Since there is no definitive test to determine whether the pericardial effusion is related to ICI use or not, by combining all the aforementioned data with the patient's positive response to steroid therapy, pembrolizumab therapy was considered the most likely underlying cause of the hemorrhagic pericardial effusion.

As a result of a comprehensive literature review, we have identified 11 reports of malignancy-negative hemorrhagic pericardial effusion attributed to the use of ICIs, as outlined in Table 2 [7-14].

**Table 2:** Reviewed 11 cases and our case PDL-1: programmed cell death ligand 1; CTLA-4: cytotoxic T-lymphocyte-associated antigen 4; PD-1: programmed cell death receptor 1

Author	Year	Journal	Age (years)	Sex	Smoker	Time of onset (days)	Type of ICI
Altan et al. [7]	2019	J Thorac Oncol	72	Male	Yes	78	PDL-1
Altan et al. [7]	2019	J Thorac Oncol	65	female	Yes	131	PDL-1 and CTLA-4
Altan et al. [7]	2019	J Thorac Oncol	57	Male	Yes	98	PDL-1
Yun et al. [8]	2015	Case Rep Oncol Med	59	Male	No	147	CTLA-4:ipilimumab
Saade et al. [9]	2019	J Immunother Cancer	58	female	Yes	42	PD-1:nivolumab
Saade et al. [9]	2019	J Immunother Cancer	65	male	Yes	484	PD-1:nivolumab
Naime et al. [10]	2018	Journal of Cancer Therapy	52	male	Yes	42	PD-1:nivolumab
Uczkowski et al. [11]	2023	Clin Case Rep	54	male	No	59	PD-1:pembrolizumab
Harada et al. [12]	2020	Thorac Cancer	63	female	No	63	PD-1:pembrolizumab
Nesfeder et al. [13]	2016	Int J Cardiol	64	male	Yes	119	PD-1:nivolumab
de Almeida et al. [14]	2018	J Immunother	69	male	Yes	334	PD-1:nivolumab
Our Case	2023	N/A	44	female	No	390	PD-1:pembrolizumab

This exclusion was necessary since the presence of malignancy in the pericardial fluid could potentially confound data interpretation. Tables 3-7 represent a summary of the key characteristics observed in these case reports.

**Table 3:** Type and stage of cancer S-NSCLC: squamous-cell carcinoma non-small cell lung cancer; A-NSCLC: adenocarcinoma non-small cell lung cancer

Type and stage of cancer	Prior cases n=11	%	Our case	Total cases n=12	%
S-NSCLC	1	9	0	1	8
A-NSCLC	9	82	0	9	75
Melanoma	1	9	0	1	8
Lymphoma	0	0	1	1	8
IIIb	2	18	0	2	17
IV	9	82	1	10	83

**Table 4:** Molecular profile of cancer KRAS: kirsten rat sarcoma viral oncogene homolog; BRAF: v-raf murine sarcoma viral oncogene homolog B; PD1: programmed cell death receptor 1; PI3KCa: phosphatidylinositol-4,5-bisphosphate 3-kinase catalytic subunit alpha; PTEN: phosphatase and tensin homolog; EGFR: epidermal growth factor receptor; HER2: human epidermal growth factor receptor 2; CD79a: cluster of differentiation 79a

Molecular profile of cancer	Prior cases n=11	%	Our case	Total cases n=12	%
KRAS	3	27	0	3	25
BRAF	2	18	0	2	17
PD1	2	18	0	2	17
PI3KCa	1	9	0	1	8
PTEN	1	9	0	1	8
EGFR	1	9	0	1	8
HER2	1	9	0	1	9
CD79a	0	0	1	1	8
No driver mutation	4	36	0	4	33
Not mentioned	4	36	0	4	33

**Table 5:** Treatments for cancer prior to hemorrhagic pericardial effusion R-CHOP: rituximab, cyclophosphamide, doxorubicin, vincristine, prednisone; RICE: rituximab, ifosfamide, carboplatin, etoposide; CAR-T: chimeric antigen receptor T-cell; PDL-1: programmed cell death ligand 1; PD-1: programmed cell death receptor 1; CTLA-4: cytotoxic T-lymphocyte-associated antigen 4

Previous treatments	Prior cases n=11	%	Our case	Total cases n=12	%
None	2	18	0	2	17
Radiation therapy	5	45	1	6	50
Cisplatin	3	27	0	3	25
Carboplatin	7	64	0	7	58
Pemetrexed	8	73	0	8	67
Bevacizumab	2	18	0	2	17
R-CHOP	0	0	1	1	8
RICE	0	0	1	1	8
CAR-T cell therapy	0	0	1	1	8
PDL-1 inhibitor	3	27	0	3	25
PD-1 inhibitor	7	64	1	8	67
CTLA-4 inhibitor	2	18	0	2	17
Nivolumab	5	45	0	5	42
Ipilimumab	1	9	0	1	8
Pembrolizumab	2	18	1	3	25

**Table 6:** Treatments and interventions for hemorrhagic pericardial effusion NSAIDs: non-steroidal anti-inflammatory drugs; ICIs: immune checkpoint inhibitors

Treatment	Prior cases n=11	%	Our case	Total n=12	%
Pericardiocentesis	8	73	1	9	75
Pericardial window	5	45	0	5	42
Corticosteroids	6	55	1	7	58
NSAIDs	1	9	0	1	8
ICIs discontinued permanently	6	55	1	7	58
ICIs discontinued and resumed	3	27	0	3	25

**Table 7:** Outcomes of hospitalization

Outcome	Prior cases n=11	%	Our case	Total n=12	%
Cancer progression	2	18	0	2	17
Resolution of effusion	6	55	1	7	58
Discharge	9	82	1	10	83
Death	2	18	0	2	17

In Table 3, it is evident that the most common malignancy associated with the occurrence of hemorrhagic pericardial effusion is lung cancer, which was observed in 83% of cases. This finding is consistent with previous research that has established a link between pericardial disease and lung cancer [5,15]. Among the identified cases of hemorrhagic pericardial effusion, 67% of subjects were treated with PD-1 inhibitors. One hypothesis suggests that a history of radiation therapy in lung cancer may potentially contribute to the development of pericardial effusion in addition to ICI use [4,16]. However, as depicted in Table 5, only 50% of patients received radiation therapy.

Variable onset of ICI-induced pericardial disease has been reported. Specifically, for pembrolizumab, based on the pharmacovigilance analysis, the median time of onset is reported to be 21 days [5]. However, the majority of sources indicate that the onset typically occurs within the first three to six months of treatment [15]. The median time of the development of pericardial disease after the initiation of ICIs was 165 days for our study subjects.

The first report of pembrolizumab-associated cardiac tamponade was described in 2018 managed by pericardial drainage and steroids [17]. Pericardial disease has been observed in approximately 0.3% of all reported adverse events related to ICI therapy [18]. Among patients receiving ICIs, the incidence of pericardial disease is 1.5%, while tamponade occurs in around 0.7% of cases [15]. Reliable data regarding the incidence of hemorrhagic pericardial effusion are currently unavailable.

The mechanism of ICIs is closely related to the inhibition of the interaction between PD-1 and programmed cell death ligand 1 (PDL-1). This blockade prevents apoptosis and stimulates T cells, ultimately resulting in the destruction of cancer cells. However, the overactivation of T cells can also target healthy tissues, leading to the development of irAEs. Although the exact mechanism of T cell-mediated cardiovascular irAEs remains incompletely understood, a widely accepted theory suggests that it involves antigenic cross-reactivity between tumor and cardiac cells [19].

The most common clinical presentations of cardiac tamponade include shortness of breath, chest pain, respiratory failure, and hemodynamic collapse in severe cases [20].

The diagnosis of cardiac tamponade is established based on a comprehensive assessment that includes the medical history, a thorough physical examination, ECG, and, most importantly, cardiac imaging. ECG alterations often involve low-voltage QRS, ST/T wave abnormalities, and electrical alternans. Transthoracic echocardiography is a valuable tool for identifying the presence of pericardial effusion, with or without signs of tamponade physiology. CT scan or MRI can provide additional information about characteristic pericarditis-type changes.

The treatment of ICI-associated toxicities depends on the severity of the presentation. Cardiotoxicity is categorized into four grades. Grade 1 patients have abnormal cardiac biomarkers and are asymptomatic with normal ECG results. Grade 2 cases also have abnormal cardiac biomarkers with mild symptoms or new ECG abnormalities. Grade 3 individuals present with abnormal cardiac biomarkers with moderate symptoms or new conduction delay, and Grade 4 entails moderate to severe decompensation with life-threatening complications. For Grade 2 cardiotoxicities, temporary discontinuation of ICIs is recommended along with consideration for steroid administration. In Grade 3 and 4 cases, the recommendation is to discontinue ICIs permanently and initiate high-dose steroid therapy [21]. The treatment of cardiac tamponade typically involves pericardial drainage in conjunction with steroid therapy [20].

Increased awareness of late-onset cardiac tamponade associated with pembrolizumab use may lead to timely identification of the complication and prompt implementation of essential measures including pericardial drainage, discontinuation of the causative medication, initiation of steroid therapy which all collectively may improve outcomes.

## Conclusions

In conclusion, the increasing utilization of ICIs emphasizes the importance of healthcare professionals' awareness of irAEs. In particular, timely identification and intervention in the case of cardiac tamponade, a critical and potentially fatal complication, is imperative. In our review, among ICIs, cases of hemorrhagic pericardial effusion were more commonly observed with the use of PD-1 inhibitors. Additionally, the occurrence of late-onset hemorrhagic pericardial effusion related to ICIs is an extremely rare complication that requires further investigation to elucidate predisposing factors and underlying mechanisms.
